# Some considerations concerning cochlear implantation in IFACF-ORL

**Published:** 2014

**Authors:** M Hainarosie, V Zainea, S Serban, MG Georgescu, R Hainarosie, A Marinescu, G Georgescu

**Affiliations:** * ”Carol Davila” University of Medicine and Pharmacy, Bucharest, Romania; ** ”Prof. Dr. Dorin Hociota” Institute of Phonoaudiology and Functional ENT Surgery, Bucharest, Romania

**Keywords:** cochlear implant, surgery, patient selection

## Abstract

The article analyzes the patients who have received a cochlear implant at “Prof. Dr. Dorin Hociota” Institute of Phonoaudiology and Functional ENT Surgery, Bucharest, over a period of 13 years, from the beginning of this program in the year 2000. It presents the types of devices used, the particularities of the patients, the surgical techniques and the outcome, critically analyzing the complications encountered. The authors’ comments on the selection of patient protocol, surgical intraoperative challenges and cochlear implant technologies and capabilities are presented.

Cochlear implantation consists of a surgical implantation of an electrode array in the inner ear of the patients with bilateral severe to profound sensorineural hearing loss, in order to rehabilitate their hearing. The success of a cochlear implant depends on the activation, adjustments of the implant and a lot of work with the speech and language specialist [**1**].

The first step before performing cochlear implant surgery is the selection of the patient. This is done after performing complex audiological exams (classical audiometry, Brainstem Evoked Response Audiometry), imagery exams (CT and MRI), and pediatrics, neurology, ophthalmology consults, as well as abdominal ultrasound and EKG exams [**2**,**3**].

If the patient passes these tests, the patency of the nasal fossa and pharyngeal orifice of the Eustachian tube must be assured. Minimally invasive nasal and rhino pharyngeal surgery must be performed in order to solve nasal and rhino pharynx patency prior to cochlear implant surgery. Special instruments have even been designed in order to perform surgery of the rhino pharynx. 

The cochlear implant program started in our hospital in the year 2000, under the auspices of the Romanian Ministry of Health.

The cochlear implant candidates’ cases were debated in “Prof. Dr. Dorin Hociota” Institute of Phonoaudiology and Functional Head and Neck Surgery, in Bucharest (IFACF-ORL) by the cochlear implantation team. The team was made up of the otology surgeon, anesthesiologist, audiologist, pediatrician, radiologist, speech therapist and psychologist.

The aim of this paper is to perform a retrospective study of the cochlear implant program in our hospital between the years 2000 to 2013.

The cases have been analyzed and the following have been taken into account: age and sex of the patients, causes of deafness, preoperative audiometric results, preoperative imagery and the hereby discovered inner ear malformations; the type of cochlear implant proposed and used in the end; activation and adjustments of the implant; audiometric postoperative tests; critical analysis of the complications.

Inclusion criteria were patients with severe-profound sensorineural deafness, who underwent cochlear implantation.

**Graph 1:**
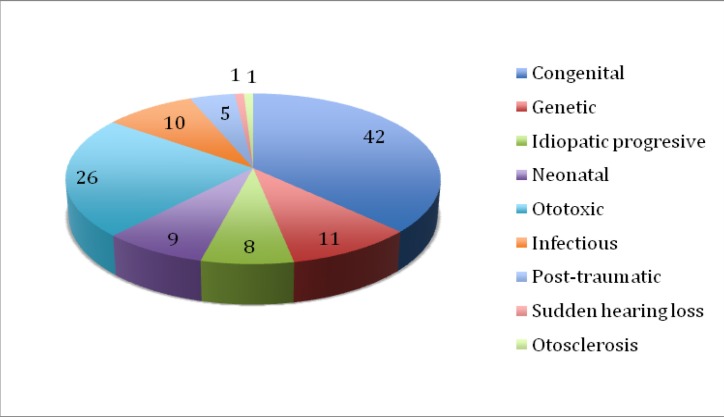
Etiology of the hearing loss

Regarding the etiology of the hearing loss, the incriminated factors were congenital, genetic, progressive idiopathic, neonatal, ototoxic, infectious, post-traumatic, sudden hearing loss and otosclerosis.

**Graph 2:**
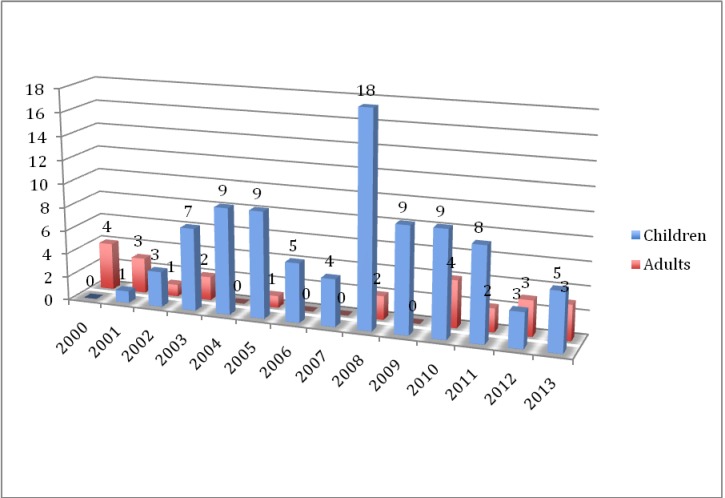
The age of the implanted patient

Concerning the age of the patients, at the beginning of the cochlear implantation program for the first two years, only adults have been implanted. After that, the main concern was to implant as many children as possible. **Graph 2** presents the evolution of the patient age.

On the radiologic exams and intraoperatively, the following anomalies and malformations of the inner ear have been found: facial canal dehiscence, facial canal providence, cochlear fibrosis, ossified cochlea, Mondini malformation.

**Graph 3:**
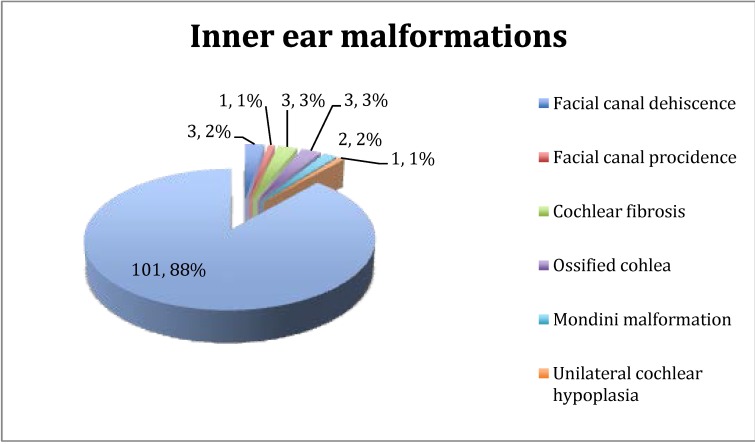
Inner ear malformations detected on imagery

The cochlear implant devices used were produced by the top three cochlear implant manufactures: MedEl Corporation (Austria), Cochlear Limited (Australia) and Advanced Bionics (USA).

**Graph 4:**
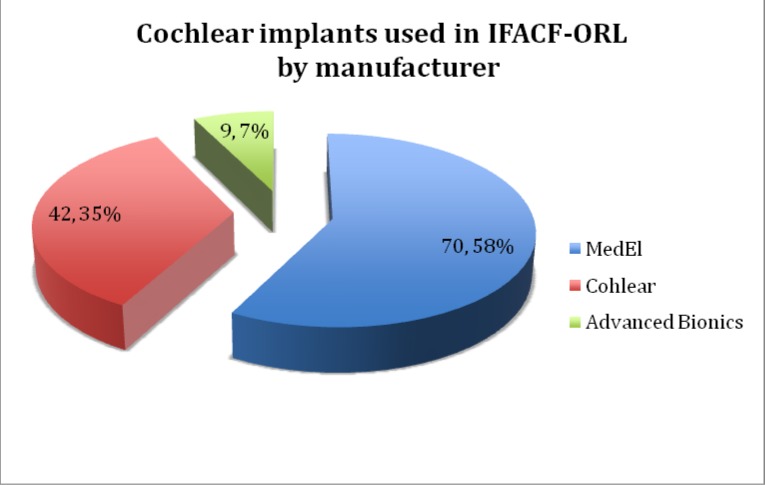
The cochlear implant manufacturers used in IFACF-ORL

Until 2004, the only cochlear implant device available on the Romanian medical market was MedEl Combi 40+. Cochlear Ltd cochlear implant device appeared in 2004 and the Nucleus 24k implant started to be used. In 2009, the first Advanced Bionics HiRes 90k cochlear implant was implanted.

**Graph 5:**
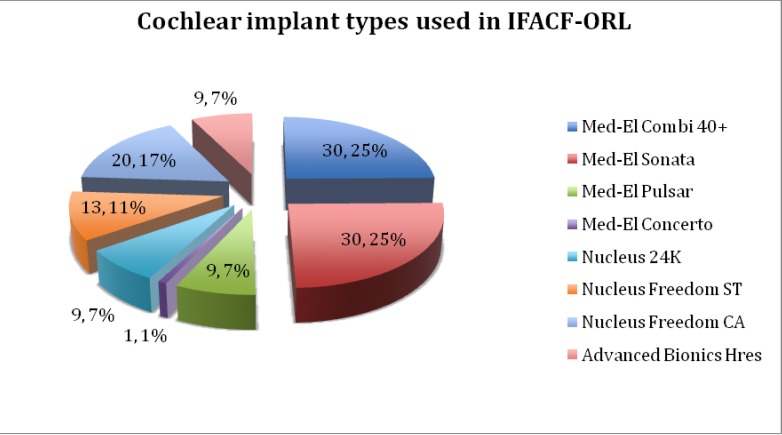
The cochlear implant manufacturers used in IFACF-ORL

**Graph 5** presents the types of cochlear devices used in our clinic. Four types of devices from MedEL company were implanted: Combi40+, Sonata, Pulsar and Concerto. Three types of devices from Nucleus company have been implanted: Nucleus 24k, Nucleus Freedom ST (Straight) and Nucleus Freedom CA (Contour Advanced). Only one type from Advanced Bionics has been implanted - Advanced Bionics HiRes 90k.

The surgical approaches that have been used are the following: mastoidectomy in 109 cases, combined in 5 cases and suprameatal in 1 case. 

The insertion of the electrode was done in 105 cases via cochleostomy and in 10 cases through the round window. In 94 cases, the insertion of the electrode was complete and in 21 cases, the insertion was incomplete, but functional. The maximum number of electrodes that remained outside the cochlea was 4.

The complications encountered intraoperatively were the following: external auditory canal lesions (2 cases); lesions of the tympanic membrane (2 cases); perilymphatic gusher (3 cases); dry labyrinth (2 cases); facial nerve electric stimulation from the implant (2 cases) and hemorrhagic complication (1 case). All the intraoperative complications were solved on the spot. 

**Graph 6:**
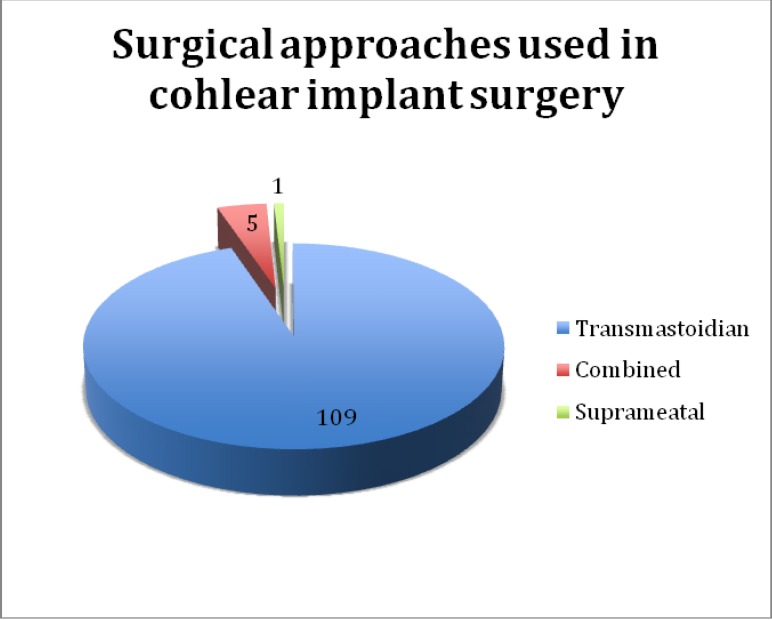
Surgical approaches used for cochlear implantation

We have performed a number of 8 re-interventions (9%) due to: cochlear implant failure (5 cases); trauma (1 case); meningitis (1 case); cholesteatoma (1 case).

All five cases of cochlear implant failure were MedEl first generation implant. The failure of the device took place between 4 and 9 years after implantation. The company replaced the devices with a newer generation, Pulsar.

Our results are the following: 31 of our children attended normal program kindergarten; 17 children attended a normal program school; 4 of our children attended a special school for children with hearing disorders; 13 children attended a normal middle school and 6 children a middle school for children with hearing disorders. 13 children attended a normal high school and only 1 child attended a special hearing disorders high school. Two of our children went to normal program university studies; two of the children left the country after high school and learned two foreign languages. 

In conclusion, the experience concerning the cochlear implantation in the institute is growing. Currently, three different cochlear technologies, from different manufactures, can be implanted. Each technology has its own particularity regarding the surgical procedure and the adjustments of the device. The goal is to minimize the age of the deafness detection in children and to implant the child at an early age. We are also trying to educate our fellows and colleagues in avoiding the use of ototoxic drugs in the pediatric population.
